# Coronary CT radiation dose reduction strategies at an Australian Tertiary Care Center – improvements in radiation exposure through an evidence‐based approach

**DOI:** 10.1002/jmrs.358

**Published:** 2019-11-06

**Authors:** Christian R. Hamilton‐Craig, Karman Tandon, Bianca Kwan, Karen DeBoni, Chris Burley, Allan J. Wesley, Rachael O’Rourke, Johanne Neill, Kelley R. Branch

**Affiliations:** ^1^ The Prince Charles Hospital Brisbane Queensland Australia; ^2^ University of Queensland Brisbane Queensland Australia; ^3^ Griffith University School of Medicine Sunshine Coast Queensland Australia; ^4^ University of Washington Seattle Washington USA

**Keywords:** cardiovascular computed tomography, coronary CT, education, prospectively triggered coronary CT, radiation dose, tube voltage

## Abstract

**Introduction:**

Coronary CT Angiography (CCTA) is a rapidly increasing technique for coronary imaging; however, it exposes patients to ionising radiation. We examined the impact of dose reduction techniques using ECG‐triggering, kVp/mAs reduction and high‐pitch modes on radiation exposure in a large Australian tertiary CCTA service.

**Methods:**

Data on acquisition modes and dose exposure were prospectively collected on all CCTA scans from November 2009 to March 2014 at an Australian tertiary care centre. A dose reduction algorithm was developed using published techniques and implemented with education of medical staff, radiographers and referrers. Associations of CCTA acquisition to radiation over time were analysed with multivariate regression. Specificity in positive CCTA was assessed by correlation with invasive coronary angiography.

**Results:**

3333 CCTAs were analysed. Mean radiation dose decreased from 8.4 mSv to 5.3, 4.4, 3.7, 2.9 and 2.8 mSv (*P* < 0.001) per year. Patient characteristics were unchanged. Dose reduction strategies using ECG‐triggering, kVp/mAs reduction accounted for 91% of the decrease. High‐pitch scanning reduced dose by an additional 9%. Lower dose was independently related to lower kVp, heart rate, tube current modulation, BMI, prospective triggering and high‐pitch mode (*P* < 0.01). CCTA specificity remained unchanged despite dose reduction.

**Conclusion:**

Implementation of evidence‐based CCTA dose reduction algorithm and staff education programme resulted in a 67% reduction in radiation exposure, while maintaining diagnostic specificity. This approach is widely applicable to clinical practice for the performance of CCTA.

## Graphical abstract

Cardiac CT angiography dosing was reviewed after implementation of dose reduction strategies. 3333 CCTAs were analyzed. Implementation of evidence‐based dose reduction strategies and a decision‐support algorithm accounted for 91% of dose reduction, with 9% attributable to hardware. Significant reductions in radiation exposure are feasible in real‐world clinical practice and can be successfully applied through better education and training.

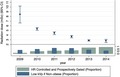

## Introduction

Coronary computed tomographic angiography (CCTA) has emerged as an important diagnostic imaging modality for the assessment of coronary artery disease (CAD) and has now entered both European and American guidelines.^1^ CCTA is appropriate for diagnosis and risk assessment in patients with low to intermediate CAD risk, who also tend to be younger and at higher risk of cancer from ionising radiation exposure. Prior to widespread introduction of radiation dose reduction techniques, CCTA studies had an average effective dose of 15.7 mSv, with some studies reaching up to 20 mSv or more.^2^

Radiation exposure during CCTA was first highlighted by the seminal *Prospective Multicentre Study On Radiation Dose Estimates Of Cardiac CT Angiography In Daily Practice* (PROTECTION) studies, which reported wide variability in radiation doses. Dose reduction techniques such as prospective ECG‐triggered scanning, aggressive heart rate control, decreased tube voltage for non‐obese patients, tube current modulation and high‐pitch scanning reduce radiation exposure and maintain image quality.^3^, ^4^, ^5^ However, few studies to date demonstrate implementation dose reduction strategies effectiveness during clinical application in a large, real‐world cohort.

This study examined the impact of a CCTA dose reduction programme on average CCTA radiation dose at a large Australian academic centre over 4.5 years. We assessed the associations of CCTA and patient parameters to reductions in CCTA radiation dose after application of evidence‐based dose reduction strategies based on the succession of PROTECTION studies.

## Methods

### Study population and patient characteristics

All sequential CCTA scans ordered for native coronary artery evaluation between November 2009 and March 2014 at a large academic single centre in Australia were included (The Prince Charles Hospital, Brisbane, Queensland, Australia). Scanning indications included symptoms of stable coronary disease in low‐ to intermediate‐risk patients, exclusion of coronary anomalies and preparation for non‐coronary cardiac surgery (as described in Medicare Item number 57360). Data were prospectively entered at the time of imaging by the attending radiographer and analysed in a retrospective cohort design. Non‐diagnostic scans were excluded (more than 1 uninterpretable coronary segment <1.5 mm in diameter, CADRADS = N). This study was approved by the Institutional Human Research Ethics Committee (HREC/15/QPCH/129) and was HIPAA compliant. Studies after March 2014 are being followed as part of a separate HREC protocol.

### Educational programme

**Figure 1:**
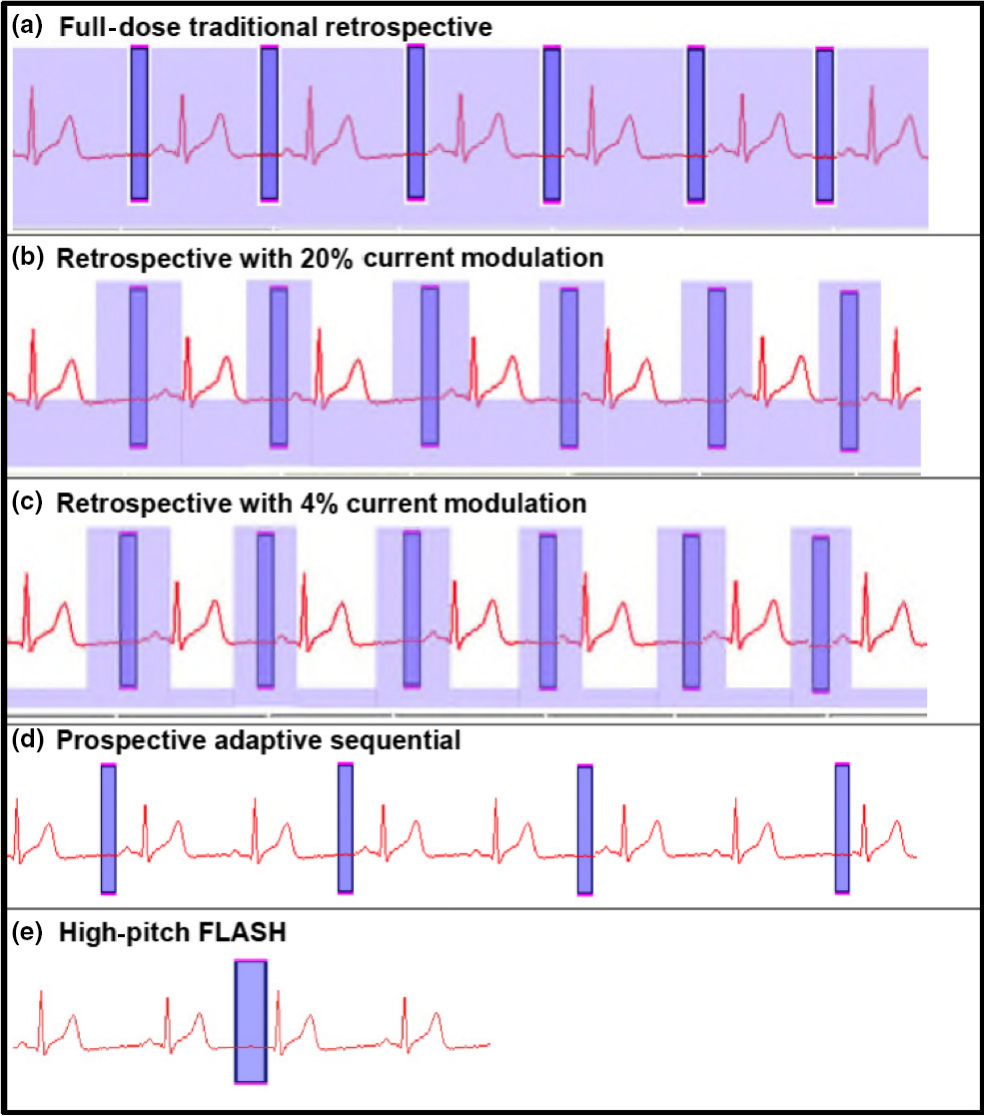
Radiation Exposure during various scan modes. Light blue corresponds to tube current‐related radiation exposure in retrospective scans (Panels A–C). Tube current modulation reduces exposure between acquisition windows (Panels B, C). Dark blue corresponds to the acquisition window itself. Prospective scanning causes radiation exposure only during acquisition (Panels D, E).

From 2010, imaging physicians and technologists participated in a formal education programme that included weekly lectures, weekly journal clubs on radiation dose reduction techniques, monthly radiation dose audits by scan indication, quarterly ‘cath‐correlation’ meetings comparing diagnostic performance of CCTA and invasive catheter angiograms, and biannual grand rounds symposia that reviewed each year’s advances in CCTA technology and institutional performance benchmarked against international standards as defined by the Society for Cardiovascular Computed Tomography (SCCT). A decision support algorithm based on published evidence was developed to assist in the appropriate selection of scan parameters for CCTA (Fig. 1). Quarterly quality‐assurance meetings assessed CCTA specificity against invasive angiography.

### Imaging protocol

CCTA was performed on dual‐source systems, either a first‐generation 64‐slice Siemens Definition CT Scanner (Somaton Definition, Siemens Medical, Erlangen, Germany), with 330 ms rotation time and a flying Z‐spot with 0.6 mm collimation, or a second‐generation Siemens Definition Flash 128‐slice scanner with 280 ms rotation time. All patients received sublingual nitroglycerin 0.4 mg and oral or intravenous beta‐blockade prior to scanning. Iodinated contrast was dispensed through a Covidien (North Ryde, New South Wales, Australia) dual‐phase injector (80–100 mL Visipaque at 5–6 mL/sec delivered through an 18‐guage cannula in the cubital fossa followed by 80 mL normal saline).

CCTA were acquired using the following commercially available ECG‐gating methods (Fig. 1 panels A–E).

1. Retrospective ECG‐gated spiral acquisition without tube current modulation (TCM) (‘full‐dose’ scan) (Fig. 1A).
2. Retrospective ECG‐gated spiral acquisition with (TCM) to 20% outside the data acquisition window (Fig. 1B).
3. Retrospective ECG‐gated spiral acquisition with tube current modulation to 4% outside the data acquisition window (‘*Min‐Dose* ^®^’ Fig. 1C).
4. Prospective ECG‐triggered axial acquisition at 70% of the R‐R interval with an automated narrow data acquisition window (‘*Adaptive Sequential* ^®^ *’,* Fig. 1D).
5. High‐pitch spiral (pitch 3.4) single‐heartbeat acquisition (‘*FLASH* ^®^ *’* mode, Fig. 1E).

#### Dose reduction

**Figure 2:**
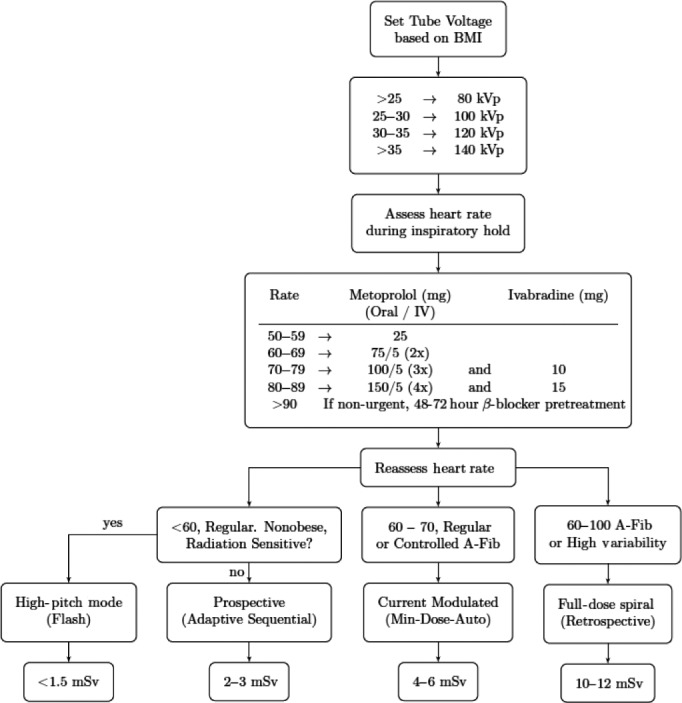
CCTA Protocol Algorithm. HRV, heart rate variability; TCM, tube current modulation; WDAW, widened data acquisition window to 30–70% of RR‐interval.

Prior to this study, the choice of ECG‐gating method, tube voltage, tube current modulation and heart rate control pre‐medication was at the discretion of the imaging medical personnel, with no specific decision support tool. These staff included: ANZCTCA Level B/ SCCT‐Level 3 accredited imaging specialists (either radiologist or cardiologist), an imaging fellow (an MD/clinician) who supervises each CCTA acquisition in person and the duty radiographers. During the study period, staff were educated to implement evidence‐based dose reduction techniques including; heart rate target of <60 bpm; preferential use of prospectively gated scanning; tube voltage reduction from ‘standard’ 120 kVp to 80‐100 kVp when scanning non‐obese patients; tube current modulation when heart rate was high or irregular; and use of high‐pitch ‘Flash’ mode after August 2012 (installation of a second‐generation dual‐source scanner). These interventions were organised into a decision support algorithm tool (Fig. 2) in order to facilitate appropriate choice of scanner acquisition mode for each individual patient and then applied prospectively for data collection. Iterative reconstruction (IR) algorithms were standard on the Siemens Definition Flash scanner (Sinogram Affirmed Iterative Reconstruction ‘SAFIRE™’) and were standardised at a ‘SAFIRE™’ level of 2 for all studies. Use of IR does not reduce photons and radiation exposure per se, but rather to enable noise reduction on images at lower exposure factors than standard kVp/mAs, thus reducing dose.^6^ Since we did not change CT acquisition parameters based on the availability of IR, the use of IR was not included in the regression analysis.

#### Dose

Dose‐length products (DLP) obtained from the CT console were used to calculate the effective radiation dose for each scan using the conversion factor 0.014 mSv/(mGy cm).^7^ All data were entered prospectively and tabulated by month over the study period.

### Diagnostic accuracy

CCTA diagnostic specificity was determined by comparison to invasive catheter angiography. Only specificity could be analysed before and after the mid‐point of the study to allow comparison of an ‘early’ and ‘late’ cohort during the dose reduction intervention period. Sensitivity was not assessed because only patients with positive CCTA (>50% stenosis) were clinically referred for invasive coronary angiography. Data were verified using the hospital Agfa Heartlab (Greenville, South Carolina) database with ethics/IRB approval. The results of invasive coronary angiography were considered to be ‘gold standard’, and specificity calculated accordingly. Due to the excellent negative predictive value of CCTA (14), normal studies do not routinely proceed to invasive angiography, thus sensitivity cannot be assessed.

### Statistical analysis

Monthly point estimates of effective dose were calculated over the study period. 95% confidence intervals were calculated for each point estimate. Heart rate, prospective ECG‐triggering, tube voltage and effective dose (mSv) for years 2010–2014 were compared with 2009. Statistics were performed using STATA (StataCorp, College Station, Texas) Continuous variables were compared using linear regression and categorical variables were compared by chi‐squared analysis. Linear correlation of continuous variables was assessed with Pearson’s correlation coefficient. Linear regression was used to test associations between clinical and CT parameters and radiation dose. A *P*‐value of <0.05 was considered statistically significant. Collinearity of these variables was excluded by calculation of variance inflation factors.

## Results

The study included 3333 consecutive CCTA scans. Patient characteristics and imaging parameters are listed in Table 1. The patient population was similar with regard to gender, BMI, indication for CCTA and prevalence of coronary disease throughout the study.

**Table 1:** Annual patient demographics, scan mode, total radiation dose and β & *P* values in multivariate regression over total dose.

	2009	2010	2011	2012	2013	2014	All	β‐coeff	*P*‐value
*N*	55	873	797	793	696	119	3333		
Female (%)	na	47	47	53	50	53	50	−0.40	<0.01
BMI (kg/m^2^)	29.4 [27.2, 31.5]	29.3 [28.9,29.]	29.3 28.8,29.7]	29.2 [28.7, 29.7]	29.6 [29.1, 30.1]	29.1 [27.7, 30.5]	29.3 [29.1, 29.6]	0.15	<0.001
Heart rate	64.0 [61.1, 66.8]	63.9 [63.2, 64.7]	61.1 [60.5, 61.9]	58.3 [57.8, 58.9]	57.5 [57.0, 58.0]	57.8 [56.5, 59.0]	60.4 [60.1, 60.7]	0.02	<0.001
Total dose mSv (% reduction)	8.4 [6.6, 10.2]	5.3 [5.0, 5.6] (37%)	4.4 [4.1, 4.7] (47%)	3.7 [3.4, 3.9] (56%)	2.9 [2.7,3.0] (65%)	2.8 [2.4, 3.1] (67%)	4.2 [4.0, 4.3]		<0.001
Scan mode (%)									
Full dose			1	1			1		
20% TCM	31	9	5	1			4		
4% TCM	60	60	54	53	17	11	46		
Prospective	9	31	40	41	69	65	44		
High pitch				4	14	24	5		
Dose by scan mode mSv									
Full	12.8	12.6	12.9						
20% TCM	14.1	11.1	10.5	10.6	11.3	‐2.49	<0.001		
4% TCM	5.6	6.0	5.4	4.6	5.1	5.3	5.4	−7.54	<0.001
Prospective	7.9	2.4	2.1	2.3	2.6	3.0	2.4	−10.35	<0.001
High pitch				1.3	1.4	1.1	1.3	−10.37	<0.001
Tube voltage <120 kVp (%)	36	42	52	53	33	28	44	−1.96	<0.001

TCM, tube current modulation.

95% confidence intervals.

**Figure 3:**
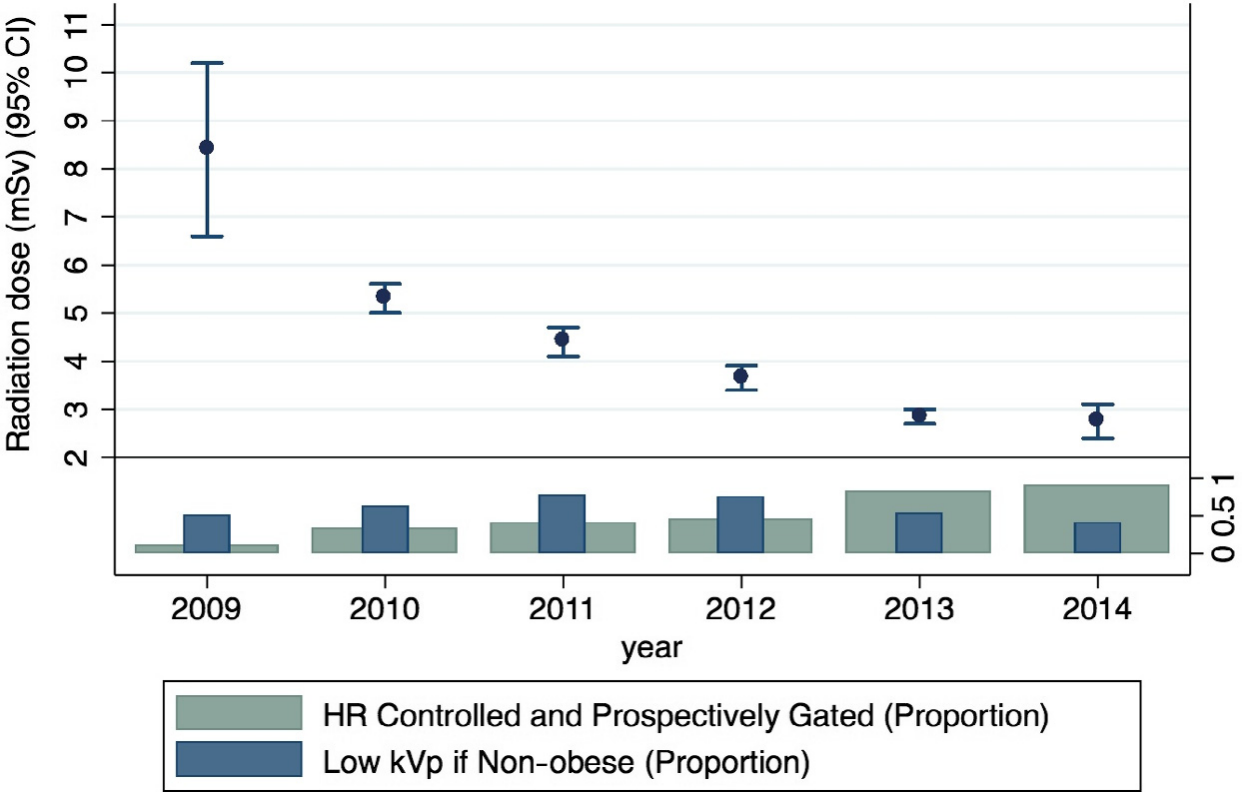
CCTA radiation dose over time compared with dose reduction strategies.

Effective dose decreased by 67% over the testing period, falling from 8.4 mSv in 2009, to 5.3, 4.4, 3.7, 2.9 and 2.8 mSv respectively (*P* < 0.00005; Fig. 3). Changes in CT acquisition protocols using the decision support algorithm (Fig. 2) accounted for 91% of total radiation dose reduction. Compared with 2009, ECG‐triggered ‘prospective’ CCTA and lower kVp were used more commonly over time (*P* < 0.0005 for trend; Table 1). Average heart rate decreased from 64.0 ± 1.4 to 57.5 ± 0.62 (*P* < 0.01). Prospective ECG‐triggered scans increased from 9% in Nov‐Dec 2009 to 89% in 2014 and accounted for 72% of the decrease in radiation dose, independent of tube voltage. Decreasing tube voltage accounted for 19% of radiation dose reduction while introduction of high‐pitch single‐heartbeat acquisition reduced dose an additional 9%.

Stepwise multivariable regression demonstrated the factors independently associated with lowering radiation dose that include lower heart rate, lower body mass index, female gender, use of prospective ECG‐triggered CCTA and lower kVp settings (*P* < 0.01 for all associations; beta‐coefficients shown in Table 1).

Figure 3 illustrates the relationship between radiation dose and the proportion of patients receiving one or more dose reduction techniques. Compared with 2009, increasing use of the prospective ECG‐triggering resulted in significant radiation dose reduction.

Specificity and diagnostic accuracy were measured in 589 patients with CCTA >50% stenosis who also underwent invasive catheter angiography. Specificity was 0.75 in the early study period and 0.76 in the later study period, which is slightly higher than the specificity reported in the 3 major international diagnostic accuracy trials of 64‐slice CCTA.^8^, ^9^, ^10^

## Discussion

This study demonstrated a 67% CCTA radiation exposure decrease during routine clinical practice through implementation of evidence‐based dose reduction programme. Staff education, including familiarity with available scan modes, the evidence for reductions in kVp, tube voltage and ECG‐triggering, and implementation of a decision support algorithm, resulted in some of the lowest mean CTCA doses reported in the literature.^11^, ^12^, ^13^ Our approach is strikingly similar to the subsequently published recommendations by the EACVI/EANM/ESCR report on strategies for radiation dose reduction.^14^ We demonstrated over 90% of CCTA radiation dose reduction can be derived from institutional standardisation of dose reduction techniques and education of imaging staff. This includes more ‘aggressive’ heart rate control which allows for prospective ECG‐triggering and high‐pitch CCTA scan modes, and reduced kVp. These two steps account for the majority of radiation dose savings. Importantly, there were no adverse events from heart rate control medication, and the safety of this strategy has previously been reported by our group.^15^

**Figure 4:**
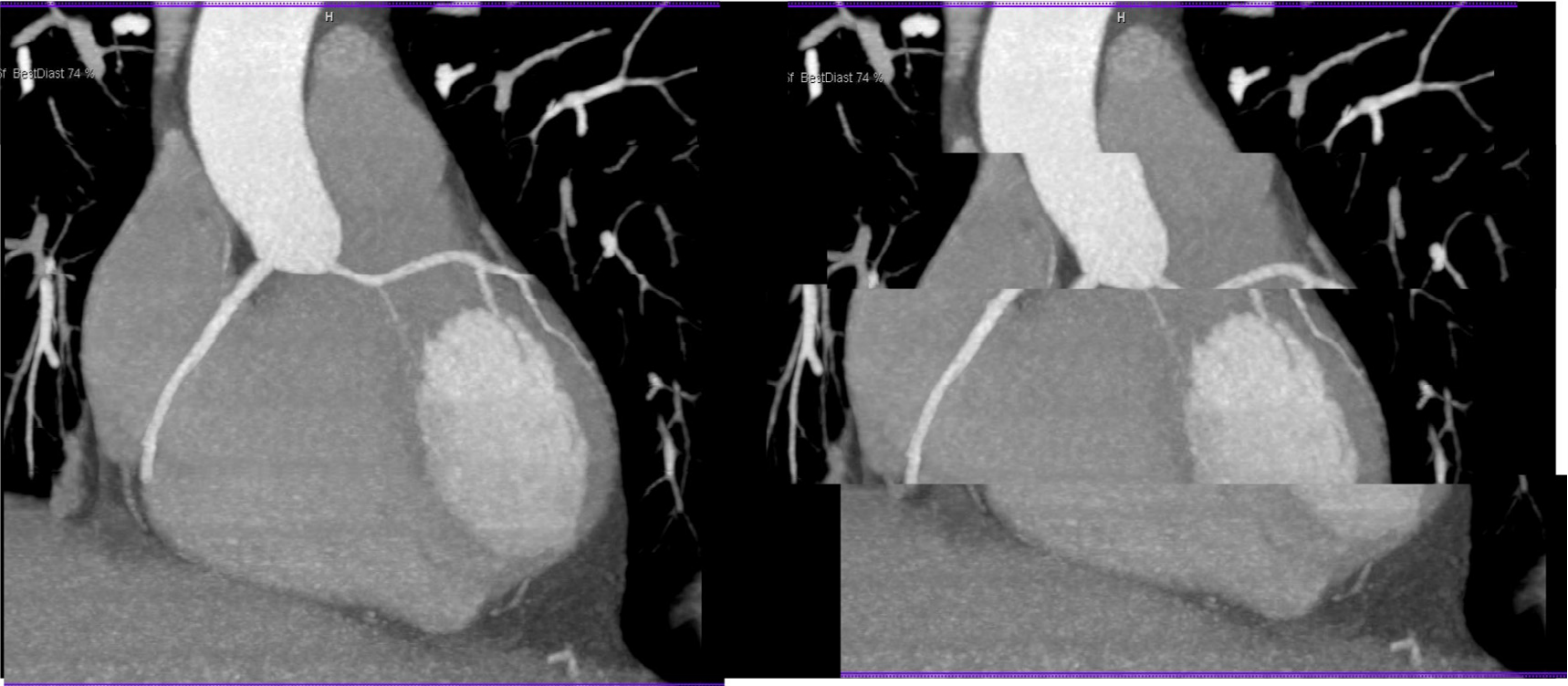
The effect of heart rate variability on a prospectively gated CCTA scan.

Early CCTA used only retrospective ECG‐gated spiral acquisition with low pitch (overlapping imaging fields), which frequently delivered radiation doses of 20 mSv or more.^16^ Improvements in software and hardware published between 2006 and 2009 allowed for axial ‘step‐and‐shoot’ imaging with prospective ECG‐triggering, without the need for imaging field overlap, that resulted in equivalent or better image quality at substantially lower dose.^17^ In prospective ECG‐triggering, an axial slice is obtained during one gantry rotation, the table moved caudally one detector width with the beam off, and this is repeated until the full *z*‐axis of the heart is covered. However, this technique requires a slow and stable heart rate to prevent misalignment and banding artefacts that can obscure the contour of the coronary vessels (Fig. 4). Our algorithm for heart rate reduction using mainly oral beta‐blockers and/or ivabradine reduced mean heart rate from 64.0 ± 1.4 bpm to 57.5 ± 0.62 bpm over the study period (*P* < 0.001). This allowed increased use of prospective ECG‐gating.

The PROTECTION‐I study in 2009 was the first to show that the radiation dose is improved by prospective triggering.^18^ PROTECTION‐I examined 685 CCTA scans at 47 institutions and identified a mean radiation dose of 11.2 mSv, driven by a predominance of retrospectively gated scans. In a smaller subset of just 99 prospectively triggered scans, a 68% dose reduction was observed. At our centre, this publication prompted education of faculty, fellows and CT staff to improve heart rate control to maximise the use of prospectively triggered CCTA. Between 2009 and 2014, average heart rate declined steadily from 64.0 to 57.5 bpm with higher dose beta‐blockade and addition of ivabradine, with a concomitant increase in prospective triggering to nearly 90% of all scans.

However, prospective triggering is not possible in patients with refractory high or irregular heart rates. In these patients, tube current modulation and decreasing tube voltage are essential to minimise radiation dose. Animal, in vitro, and small patients studies in the mid‐2000s described these techniques before they were examined in larger cohorts between 2006 and 2010.^18^, ^19^, ^20^, ^21^, ^22^ The latter studies showed consistent image quality in spite of dose reduction. In 2010, PROTECTION II demonstrated that decreased tube voltage to 100 kVp in non‐obese patients reduced radiation dose by 31% (13). Adoption of lower kVp settings in non‐obese patients in our protocol resulted in a 2.0 mSv (16%) decrease in radiation dose, a lesser degree of reduction due to the relative preponderance of prospective scans in our study compared with the PROTECTION II trial.

In our retrospective scans, tube current modulation to 4% outside of the data acquisition window (*Min‐Dose™* mode) independently reduced dose by 69% (7.5 mSv, Table 1) when compared to full‐dose scanning. Based on the studies above, and our clinical experience, when heart rate remains above 60 bpm or exhibits >10% variability we recommend the use of prospective triggering with a widened data acquisition window (‘padding’) or retrospective gating with dose modulation (Fig. 1 panels C,D).

High‐pitch (pitch 3.4) single‐heartbeat scanning reduces radiation dose and minimises imaging artefacts, but requires a newer‐generation dual‐source scanner. ‘Flash’ mode requires a heart rate of less than 59 bpm (ideally <55 bpm) to allow sufficient time between R‐R intervals for the scan to be acquired in a single heartbeat (scan time 270 ms during late diastole). After 2012, our institution installed a second‐generation dual‐source scanner, allowing us to further reduce radiation via high‐pitch scans, particularly in patients of normal body weight (enabling kVp reduction) and well‐controlled heart rates (enabling high pitch). Dose was routinely ~1 mSv in high‐pitch scans, consistent with other reports.^23^ However, similar beta‐coefficients (Table 1) for prospective‐gated and high‐pitch acquisitions, suggest that high‐pitch acquisition offers only small incremental benefit in radiation dose compared with optimisation of other factors which are routinely available on all CT scanners without the need for new hardware (heart rate, kVp, mAs, etc).

As noted above, iterative reconstruction (IR) algorithms were applied to all scans on the Siemens Definition Flash scanner (using the SAFIRE™ algorithm) but were not available on the earlier generation Siemens Definition scanner. IR is widely used as a dose reduction technique to compensate for the increased noise from lower‐dose acquisitions, but the use of IR to reduce dose was not tested in this retrospective study design since we did not vary CT acquisition parameters based on the use of IR. The use of IR as a dose reduction technique is examined in detail in the Prospective Randomized Trial on Radiation Dose Estimates of CT Angiography Applying Iterative Image Reconstruction (PROTECTION V) Study (42).

The lesson is*; *‘it's not the scanner, it's how you use it’*.* These data are applicable to the broader radiology community who can achieve substantial dose reductions through education and training in dose reduction techniques, even when using older generation CT scanners.

### Limitations

This is an observational study, rather than a randomised trial design as performed previously in the literature. However, data were prospectively collected and reflect unselected, ‘real world’ gains in radiation reduction through education and application of evidence into clinical practice, thereby increasing generalisability. This approach of clinical adoption of optimal radiation dose reduction techniques and decision support algorithms could be emulated at any institution. A second limitation is that data on beta‐blocker or ivabradine dosing were not captured, but mean heart rate fell significantly during the study period, with no adverse events. Thirdly, objective data on CCTA image quality are lacking as we did not have the resources to retrospectively evaluate image quality such as Likert scores in 3333 scans.^24^ Sensitivity could not be directly measured as patients with negative CCTA did not progress to catheterisation (appropriately). However, the specificity of CCTA remained very high compared with invasive coronary angiography and did not change over the study period^25^ and, in fact, was slightly higher than the pooled specificity (0.70) from the 3 major international diagnostic accuracy trials of 64‐slice CCTA.^8^, ^9^ Thus, it can be surmised that CCTA were of high diagnostic quality and with specificity numerically higher to that quoted in published randomised studies such as CORE‐64 and ACCURACY trials.^8^, ^10^ Lastly, all studies were completed on a single vendor system, and how single source broad‐detector technology would perform was not evaluated.

## Conclusion

Clinical application of CCTA dose reduction techniques, including optimal patient preparation with heart rate control, as well as regular education of physicians and technologists, resulted in a significant 67% reduction in radiation exposure. The majority of dose reduction (91%) was attributable to education, training, and the decision support algorithm promoting kVp reduction and improved heart rate control, thus allowing the use of prospective gating. These strategies can be applied to any CT service at a relatively low cost; upgraded hardware (high pitch) only added an additional 9% dose reduction. Standardisation of protocols and staff education are an effective near‐term solution improving the safety non‐invasive CCTA imaging in everyday clinical practice. Further studies on the impact of 4^th^ generation CT scanner infrastructure on radiation exposure are ongoing.

## Conflict of Interest

The authors declare no conflict of interest.

## Funding Information

Smart Futures Fellowship, Queensland State Government (CHC) and the Washington‐Queensland TransPacific Trust (CHC, KRB).
